# Neural-Specific Deletion of *Htra2* Causes Cerebellar Neurodegeneration and Defective Processing of Mitochondrial OPA1

**DOI:** 10.1371/journal.pone.0115789

**Published:** 2014-12-22

**Authors:** Victoria L. Patterson, Alfred J. Zullo, Claire Koenig, Sean Stoessel, Hakryul Jo, Xinran Liu, Jinah Han, Murim Choi, Andrew T. DeWan, Jean-Leon Thomas, Chia-Yi Kuan, Josephine Hoh

**Affiliations:** 1 Department of Environmental Health Sciences, Yale University School of Medicine, New Haven, Connecticut, United States of America; 2 Department of Neurology, Yale University School of Medicine, New Haven, Connecticut, United States of America; 3 Department of Genetics, Yale University School of Medicine, New Haven, Connecticut, United States of America; 4 Department of Chronic Disease Epidemiology, Yale University School of Medicine, New Haven, Connecticut, United States of America; 5 Center for Cellular and Molecular Imaging, Yale University School of Medicine, New Haven, Connecticut, United States of America; 6 Department of Pediatrics, Emory University School of Medicine and Children’s Healthcare of Atlanta, Atlanta, Georgia, United States of America; UCL Institute of Neurology, United Kingdom

## Abstract

HTRA2, a serine protease in the intermembrane space, has important functions in mitochondrial stress signaling while its abnormal activity may contribute to the development of Parkinson’s disease. Mice with a missense or null mutation of *Htra2* fail to thrive, suffer striatal neuronal loss, and a parkinsonian phenotype that leads to death at 30–40 days of age. While informative, these mouse models cannot separate neural contributions from systemic effects due to the complex phenotypes of HTRA2 deficiency. Hence, we developed mice carrying a *Htra2*-floxed allele to query the consequences of tissue-specific HTRA2 deficiency. We found that mice with neural-specific deletion of *Htra2* exhibited atrophy of the thymus and spleen, cessation to gain weight past postnatal (P) day 18, neurological symptoms including ataxia and complete penetrance of premature death by P40. Histologically, increased apoptosis was detected in the cerebellum, and to a lesser degree in the striatum and the entorhinal cortex, from P25. Even earlier at P20, mitochondria in the cerebella already exhibited abnormal morphology, including swelling, vesiculation, and fragmentation of the cristae. Furthermore, the onset of these structural anomalies was accompanied by defective processing of OPA1, a key molecule for mitochondrial fusion and cristae remodeling, leading to depletion of the L-isoform. Together, these findings suggest that HTRA2 is essential for maintenance of the mitochondrial integrity in neurons. Without functional HTRA2, a lifespan as short as 40 days accumulates a large quantity of dysfunctional mitochondria that contributes to the demise of mutant mice.

## Introduction

HTRA2 (Omi), belonging to the high-temperature requirement A (HtrA) family of stress proteins, maintains mitochondrial homeostasis in physiological conditions but also stimulates apoptosis in extreme situations [1]–[5]. Structurally, the HTRA2 protein has a central serine protease domain and a C-terminal PDZ domain that interacts and suppresses the protease activity, but loses its grasp at high temperature or after ischemic-reperfusion injury [6]. The protease activity of HTRA2 is also regulated at multiple phosphorylation sites, including phosphorylation upon activation of the p38 MAP kinase pathway in a PINK1-dependent manner [7]. Under physiological conditions, HTRA2 switches between chaperone and protease functions to prevent the buildup of misfolded proteins in the mitochondrial intermembrane space [8]. Yet, in pathological conditions, a processed form of HTRA2 is released from mitochondria to the cytosol where it binds and inhibits the activity of inhibitors of apoptotic proteins (IAPs) to accelerate cell death [9]. Loss-of-function mutations in the gene encoding *HTRA2* were found associated with Parkinson’s disease in different populations [10], [11]. However, recent studies reveal that the genetic variability in *HTRA2* differs among ethnic groups and at most only constitutes a risk factor for Parkinson’s disease [12]–[15].

One explanation to account for the lack of dominant *HTRA2* mutations in Parkinson’s disease is that HTRA2 may be indispensable for mitochondrial function [5]. Hence, only multiple subtle missense mutations of *HTRA2* have accumulated in the gene pool. This notion is supported by severe consequences in germ-line *Htra2*-null mutation and the spontaneous mouse mutant *mnd2* (motor neuron degeneration 2) that harbors a Ser276Cys missense mutation in the protease domain of *Htra2* [16]–[18]. These two mutant lines showed almost identical phenotypes, including parkinsonian symptoms, loss of striatal neurons, involution of the spleen and thymus, failure to thrive, and death before 40 days of age. Interestingly, transgenic expression of human HTRA2 in the central nervous system of *mnd2* mice prevented neurodegeneration and premature death, but also revealed accelerated aging phenotypes in the adult rescued mice, thus indicating broad systemic effects of HTRA2 deficiency [19]. However, it was uncertain until the present study whether neural-specific HTRA2 deficiency is sufficient to recapitulate the full spectrum of complex phenotypes in *Htra2*-null and *mnd2* mice.

OPA1, a large guanosine triphosphatase (GTPase) located in the inner membrane, may be an effector of HTRA2 during stress-induced mitochondrial hyperfusion, but this relationship is yet to be confirmed [20]. While fusion between mitochondrial outer membranes is mediated by two dynamin family members, Mitofusin 1 (Mfn1) and Mitofusin 2 (Mfn2) in mammals, fusion between mitochondrial inner membranes is mediated solely by OPA1 [21]. OPA1 also controls cristae remodeling and regulates the release of pro-apoptotic proteins, such as cytochrome c, into the cytosol [22]–[24]. The activities of OPA1 are regulated by proteolytic processing that generates a mixture of long and short isoforms, which are both needed for proper functions of OPA1 [25]–[28]. Past studies revealed physical interactions of HTRA2 and OPA1 in mouse brains [29], but whether HTRA2 influences the processing of OPA1 is unknown.

To assess neural-specific functions of HTRA2, we have generated *Htra2*-deficient lines from a newly created *Htra2^flox/flox^* allele to compare the phenotypes of *Htra2* deletion in the germ-line and the nervous system. Here we demonstrate that neural-specific deletion of *Htra2* results in both the neurological and non-neurological phenotypes observed upon systemic deletion. We also show that mitochondrial anomalies and defective OPA1 processing precede cerebellar neuron death in mice harboring systemic or neural-specific HTRA2 deficiency. These results reveal novel insights into the functions of HTRA2 during early postnatal brain development.

## Materials and Methods

### Animals

*Htra2^+/flox^* mice were generated by Ozgene (Perth, Australia). A FRT-flanked PGK-neomycin cassette was inserted downstream of exon 4. LoxP sites were inserted upstream of exon 2 and downstream of the selection cassette. The construct was electroporated into embryonic stem (ES) cells from C57BL/6J mice. Correctly targeted ES cells were injected into C57BL/6J blastocysts. Chimeric mice resulting from the transfer were crossed to C57BL/6J mice to generate *Htra2^+/flox-neo^* mice. These mice were crossed to OzFlpE (a knock in of *FlpE* at the *Rosa26* locus) to remove the selection cassette, followed by backcrossing to C57BL/6J to remove *FlpE*. *Htra2^flox/flox^* mice were crossed to OzCre (PGK-Cre at the *Rosa26* locus) or B6.Cg-Tg(Nes-Cre)1Kln/J (Jackson Laboratories). All animals were maintained under a 12-hour light/dark cycle, with freely available food and water. All experiments were performed in accordance with the National Institutes of Health guidelines under protocols approved by the Yale University Animal Care and Use Committee, on animals of both sexes. This study was carried out in strict accordance with the recommendations in the Guide for the Care and Use of Laboratory Animals of the National Institutes of Health. The protocol was approved by the Committee on the Ethics of Animal Experiments of Yale University (Permit Number: 2011-11214). All efforts were made to minimize suffering.

### Genotyping

Genomic DNA was extracted and PCR performed using the REDExtract-N-Amp Tissue PCR Kit (Sigma) according to manufacturer’s directions. *Htra2* alleles were determined (using 5′-ACCCACTTCTCCCCGGAGCCAGTAC, 5′-CCATCTGAAGCCACTACGAATCCT and 5′-TCCAATCACCTCCCCATCC) and the presence of Cre recombinase was assayed (using 5′-TTACCGGTCGATGCAACGAGTGATG and 5′-TTCCATGAGTGAACGAACCTGGTCG). Genotyping generated bands for wild type (WT), floxed and deleted alleles of 279 bp, 313 bp and 358 bp respectively.

### Protein extraction and Western blotting

Total cell lysates were generated in RIPA buffer (1% Nonidet P40, 0.5% sodium deoxycholate, 0.1% SDS in PBS). Protein was quantified using the Pierce BCA Protein Assay Kit (Thermo Scientific) according to manufacturer’s instructions. Westerns were performed as described [30]. PVDF was immunoblotted with antibodies against HTRA2 (R&D Systems), HSP90 or β-actin (Santa Cruz), OPA1 (mouse anti-OPA1 from BD Pharminogen, rabbit anti-OPA1 from Abcam), VDAC, MFN2, LC3β, P62 or PHB2 (Cell Signaling). ImageJ was used to quantify band intensity to calculate OPA1 processed form ratios from at least three animals per genotype.

### Pathology, histochemical staining and immunostaining

Tissues were formalin fixed and embedded in OCT for cryosectioning. H&E staining and Nissl staining were performed according to established protocols, and independently performed by the pathology core at the Tri-Institutional facility at Sloan Kettering Memorial Cancer Center. Immunostaining was performed as described [31] but delipidation was performed only for labeling with anti-proteolipid protein (JL Thomas, Yale University). Antibodies: Calbindin D-28K (Millipore), PAX6 (Developmental Studies Hybridoma Bank). Histochemical staining was performed as previously described [32] on flash frozen sections. TUNEL was performed on flash frozen tissue sections using the *In situ* Cell Death Detection Kit (Roche) according to manufacturer’s instructions. Cell death was quantified from images of appropriately matched sections from the striatum, entorhinal cortex and cerebellum, using ImageJ. At least three images per animal were quantified, with at least three animals per genotype included, and cell counts were normalized to area.

### Electron microscopy

Electron microscopy was conducted at the Electron Microscopy core facility at the School of Medicine, Yale University. Two mice per genotype per age point were perfused (4% PFA/PBS), cerebella dissected and fixed (2.5% glutaraldehyde, 2% PFA in 0.1 M sodium cacodylate, pH 7.4) overnight at 4°C. Cerebella were vibratome sectioned (200 µm) and refixed (1 hr, RT), postfixed (1% OsO4 in 0.1 M cacodylate buffer), en bloc stained (2% uranyl acetate in maleate buffer), dehydrated and embedded in Epon. Ultramicrotome sections were collected using a Leica EM Ultramicrotome and post stained with lead and uranyl acetate. Mitochondrial size was measured using ImageJ.

### Behavioral studies

For the hind limb suspension test pups were placed on a 36°C heating pad during test administration. For the weanling test battery and the grip strength test no additional heat was provided. All tests were administered to neonates at appropriate developmental ages. For all behavioral tests, pups were randomly removed from their home cage and placed into individual holding cups until testing.

The Hind Limb Suspension Test was used to evaluate proximal hind-limb muscle strength, weakness and fatigue. The test was administered daily to neonates from P4 to P10 following a previously published protocol [33]. During testing pups were suspended by their hind limbs using a 50 mL conical tube with a cotton ball placed at the bottom. On a single day of testing pups underwent two consecutive test trials: latency to fall and the number of hind limb pulls were recorded, summed over the two consecutive trials and used in statistical analysis.

The weanling observation test was used to score weanling pups for age-appropriate movements and general activity levels. The test was administered following a previously published protocol [34]. Specifically, weanling pups were observed daily from P19 to P21 for a 3-min period in a Plexiglas testing box containing a grid of 2 inch squares on the bottom. In each test session, the number of rearings, grooming events, and the number of grid lines crossed were recorded. Total activity was summed for each day and then averaged over the 3 test days for statistical analysis.

The wire mesh grip strength test was used to assess grip strength as previously described [35]. Mice were tested on alternate days from P22 to P26, during which animals were placed on a mesh grid, inverted for a maximum of 60 seconds and latency to fall was recorded. 3 trials were conducted per day, with an inter-trial interval of 5 minutes, and averaged for statistical analysis.

Statistical analysis of behavioral data was performed using IBM SPSS Statistics version 19 (SPSS, Chicago, IL). Independent T-tests were used to compare genotype differences. Outliers were removed from the data set when applicable.

## Results

### A Cre-Lox allele was designed to generate a frame-shift mutation of *Htra2* while preserving the *Loxl3* transcript

In mice, *Htra2* has 8 exons spread over approximately 3.3 Kb and is situated in a relatively gene rich region of the genome where it is transcribed from the reverse strand of DNA. The sequence that comprises exons 6–8 of the *Htra2* transcript is also transcribed in the forward direction, forming the 3′ UTR of lysyl oxidase-like 3 (*Loxl3*). To ensure all phenotypes are caused by disruption of *Htra2* expression, we generated mice with a floxed *Htra2* allele that, upon expression of Cre recombinase, deletes sequence only known to contribute to the *Htra2* transcript.

**Figure 1:**
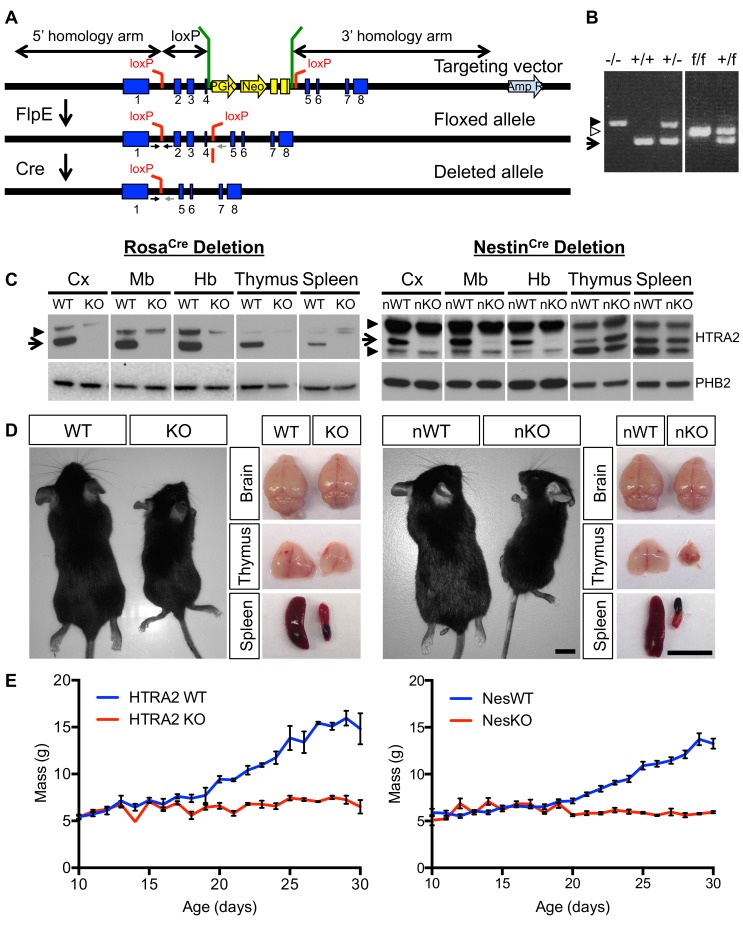
Neural deletion of *Htra2* is sufficient to generate neurological phenotypes. (*A*) Exons 2 to 4 of *Htra2* were flanked with loxP sites, with a FRT flanked neo cassette 3′ to exon 4. Expression of *FlpE* causes deletion of the selection cassette. Cre-mediated deletion causes excision of exons 2 to 4. Small arrows beneath the allele constructs denote the position of genotyping primers. (*B*) PCR from genomic DNA can distinguish WT (+, arrow, 279 bp), KO (–, filled arrowhead, 358 bp) and floxed (f, empty arrowhead, 313 bp) alleles of *Htra2*. (*C*) Western blot analysis confirmed loss of HTRA2 protein (arrow) in all tissues of HTRA2 KO mice and reduction in brain of NesKO mice (arrowheads denote non-specific bands). The levels of HTRA2 protein in NesKO spleen and thymus were comparable with NesWT. Cx: cortex, Mb: midbrain, Hb: hindbrain. PHB2 was used as a loading control. (*D*) HTRA2 KO mice and NesKO mice were smaller than WT littermates by comparison. The size of the thymus and spleen was reduced although brain was relatively normal in size (representative animals shown at P30, scale bar: 1 cm.). (*E*) Body weight of HTRA2 KO and NesKO mice did not increase beyond P18 (*n* = 56 (HTRA2 WT), 62 (HTRA2 KO), 35 (NesWT), 25 (NesKO), error bars indicate SEM).

Exons 2 to 4 were flanked with loxP sites to allow for Cre recombinase-mediated deletion (Fig. 1A). These exons encode part of the peptidase domain, including the site of the Ser276Cys mutation in *HtrA2^mnd2^* mice [17] but do not contribute to the *Loxl3* transcript. Deletion was designed to generate a frame-shift mutation and can be detected by PCR from genomic DNA (Fig. 1B).

### Germ-line deletion of *Htra2* by Rosa26-Cre caused a range of parkinsonian phenotypes

To determine whether mice with this floxed *Htra2* allele would generate parkinsonian phenotypes we first crossed *Htra2^flox/flox^* mice to mice expressing Cre recombinase from the *Rosa26* locus (OzCre, Ozgene, Australia). The resultant *Htra2^flox/flox^;Rosa26*:*Cre* mice harbor a germ-line transmissible deletion and intercrossing yielded offspring with homozygous deletion of *Htra2* in all cells (hereafter referred to as HTRA2 KO). Heterozygote animals were healthy and fertile and HTRA2 KO mice were born in the expected Mendelian ratios. Western blotting confirmed a loss of protein in all tissues, consistent with the expected ubiquitous expression of Cre recombinase (Fig. 1C).

Mice initially developed normally until P18 at which point HTRA2 KO mice failed to thrive and subsequently were smaller than WT littermates (Fig. 1D). This was apparent in the body weights of HTRA2 KO mice compared to WT; while WT mice progressively gained weight the HTRA2 KO mice did not increase in body weight past P18 (Fig. 1E). HTRA2 KO mice went on to demonstrate a progressively worsening phenotype, initially presenting at around P25 as uncoordinated movement, progressing into ataxia, loss of balance, rolling and tremors followed by lethargy. 100% of HTRA2 KO mice were euthanized by P40, due to paralysis and lack of response to stimuli. HTRA2 KO mice were also lymphopenic, exhibiting a small thymus and spleen compared to WT controls (Fig. 1D).

The phenotypes observed in HTRA2 KO mice appeared consistent with those reported for *Htra2^tm1Jdo^* and *Htra2^mnd2^* mice [16], [18], demonstrating the efficacy of the floxed *Htra2* allele upon Cre recombinase expression and validating its usefulness in exploring the contributions of specific cells and tissues to the *Htra2*-null phenotype.

### Neuronal deletion of *Htra2* by Nestin-Cre caused parkinsonian phenotypes

Having demonstrated the ability to produce the expected phenotypes using *Htra2^flox/flox^* mice, we turned to a tissue-specific Cre line to further dissect the phenotypes of HTRA2 KO mice. Considering that the *Rosa26*:*Cre* mediated knockout resulted in neurological phenotypes, we crossed *Htra2^flox/flox^* to mice expressing Cre recombinase under the control of the *Nestin* promoter to delete *Htra2* in neuronal and glial cell precursors [36].

The phenotypes observed in *Htra2^flox/flox^;NesCre* mice (hereafter referred to as NesKO mice) resembled those of HTRA2 KO mice. All heterozygotes from the neural specific deletion were healthy and fertile. NesKO mice were born in expected Mendelian frequencies, and initially developed normally. Western blotting confirmed the reduction in HTRA2 in the central nervous system and spinal cord, although some protein remains in cell types that do not express *Nestin*. No loss of HTRA2 was observed in other tissues such as spleen and thymus (Fig. 1C).

HTRA2 NesKO mice exhibited a failure to gain weight from P18 onwards, and consequently were smaller than NesWT (*Htra2^+/flox^;NesCre*) littermates (Fig. 1D–E). The initial failure to thrive was followed by the development of neurological symptoms from P25 onwards: ataxia, tremors, loss of balance, rolling and lethargy. 100% of NesKO mice were euthanized by P35, due to paralysis and lack of response to stimuli. Despite the intact expression of *Htra2* in tissues other than the nervous system, NesKO mice also present a small thymus and spleen (Fig. 1D), despite the presence of HTRA2 in immune tissues.

### Behavioral defects can be detected as early as P7 in mice lacking HTRA2

At approximately P18 HTRA2 KO and NesKO weanling pups start to display an overt phenotype. We hypothesized that a subtler phenotype may be present earlier in development then P18. To evaluate this hypothesis we conducted neonatal behavioral tests that measured muscle strength (hind-limb suspension) and activity levels (weanling observation) prior to the onset of the overt neurological phenotypes, in addition to assessing grip strength at a later point to confirm that NesKO mice recapitulate the HTRA2 KO phenotype.

**Figure 2:**
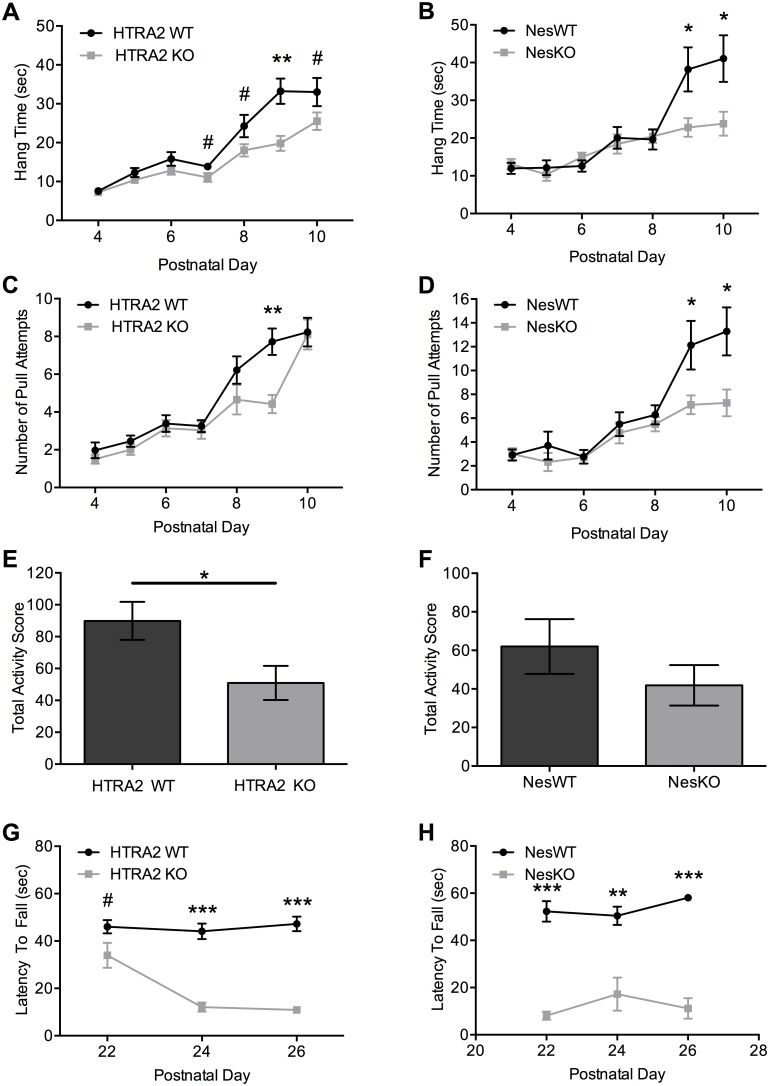
Muscle strength and total activity are decreased in *Htra2*-deficient mice. (*A–D*) The hind limb suspension test was performed on HTRA2-deficient neonates. HTRA2 KO mice performed poorly compared to WT littermates in hang time (*A*) and number of pull attempts (*C*) from P7. NesKO neonates performed poorly compared to NesWT littermates in hang time (*B*) and number of pull attempts (*D*) from P8 (n = 28 (HTRA2 WT), 19 (HTRA2 KO), 10 (NesWT), 16 (NesKO)). (*E–F*) The weanling observation total activity score was reduced in P19–21 HTRA2 KO animals (*E*) compared to WT littermates, and NesKO animals (*F*) compared to NesWT littermates (n = 19 (HTRA2 WT), 12 (HTRA2 KO), 7 (NesWT), 4 (NesKO)). (*G–H*) Grip strength was reduced in HTRA2 KO (*G*) and NesKO (*H*) animals compared to respective WT littermates (n = 17 (HTRA2 WT), 9 (HTRA2 KO) 11 (NesWT), 5 (NesKO)). Data represents Mean ± SEM (#: 0.05≤p≤0.10, *: p≤0.05, **: p≤0.001, ***: p≤0.0001 by independent t-tests).

In the hind limb suspension test HTRA2 WT, HTRA2 KO, NesWT and NesKO neonates all showed an age related increase in muscle strength demonstrated by both an increased latency to fall (Fig. 2A–B) and an increased number of hind limb pulls (Fig. 2C–D). However, both HTRA2 KO and NesKO neonates showed a decrease in muscle strength performance when compared to their respective WT littermates. Significant differences were observed in hang time and in the number of pull attempts between HTRA2 WT and KO neonates (Fig. 2A, C). Specifically, HTRA2 KO mice, when compared to their WT littermates, displayed a consistently shorter hang time and decreased number of hind limb pull attempts beginning on P7 and continuing to P10. NesKO neonates showed a similar trend (Fig. 2B, D) to the HTRA2 KO mice, with obvious differences between NesWT and NesKO neonates beginning at P8 and continuing to P10.

During the weanling observation test, HTRA2 KO weanling pups displayed significantly less total activity from P19–P21 (Fig. 2E) when compared to WT animals. NesKO animals showed a similar trend although significance was not reached (Fig. 2F). As previously reported [35], grip strength was decreased for HTRA2 KO animals compared to WT littermates at P22–P26 (Fig. 2G). The performance of NesKO animals was also significantly poorer on all days tested (Fig. 2H).

These results reveal that the loss of HTRA2 in the nervous system significantly affects not only late but also early neonatal development, as NesKO animals recapitulate both early and late phenotypes of HTRA2 KO mice. Further, these data suggest that earlier time points in neonatal development should be evaluated in future studies to help elucidate the specific role of HTRA2 in the development of observed phenotypes.

### Cell death was detected in discrete regions of *Htra2*-deleted brain

**Figure 3:**
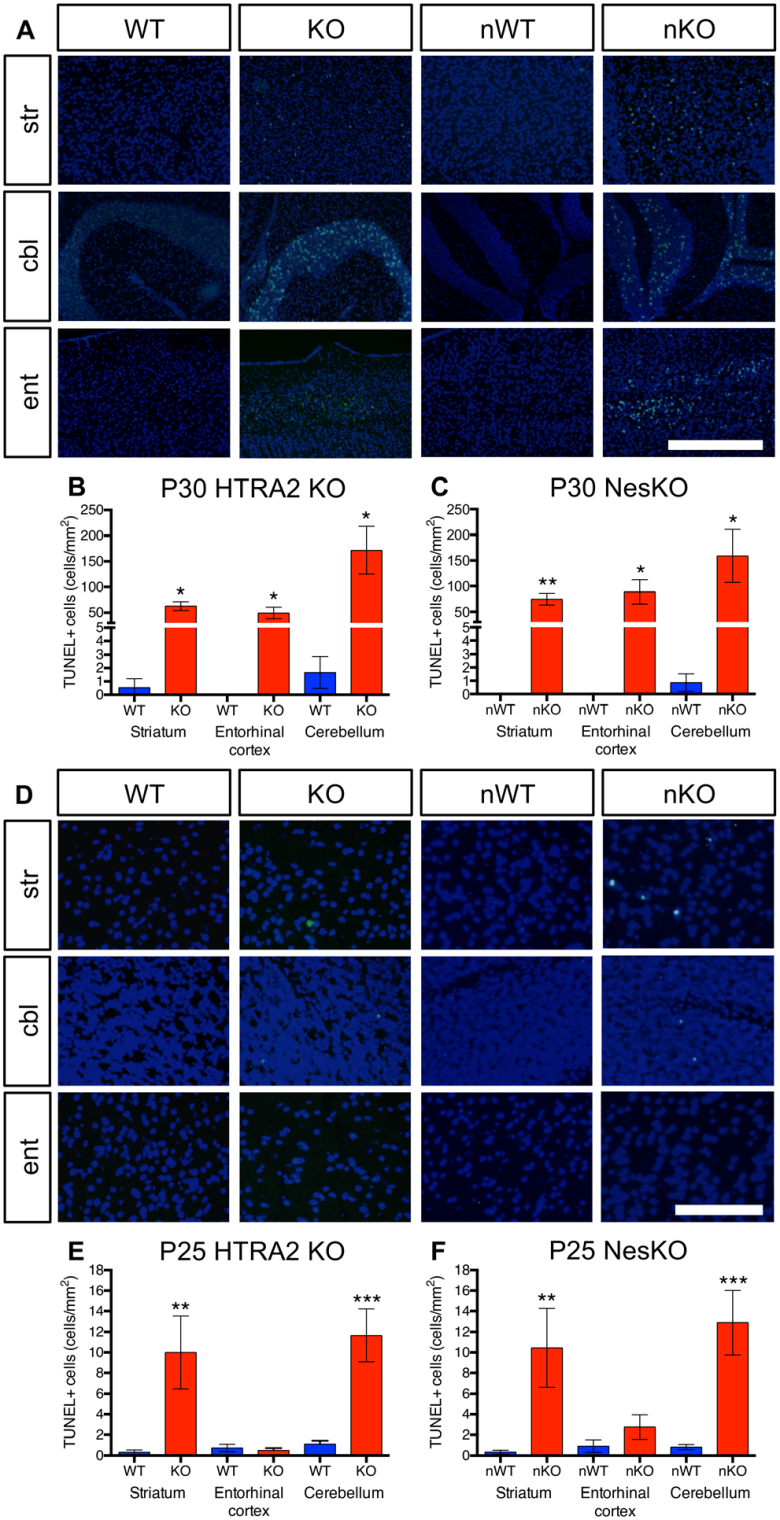
HTRA2-deficient mice exhibited increased cell death in discrete brain regions. (*A*) TUNEL staining (green) demonstrated that cell death was increased in three discrete regions of the brain in both HTRA2 KO and NesKO mice. Increased cell death was detected in the striatum (str), cerebellum (cbl) and entorhinal cortex (ent,) of P30–35 mice (n≥4 per genotype). Cell nuclei were counterstained with DAPI (blue). (*B–C*) Semi-quantitative analysis of TUNEL positive cells in HTRA2 KO and NesKO brains revealed the prevalence of TUNEL positive cells was significantly increased in striatum, entorhinal cortex and cerebellum of HTRA2 KO (*B*) and NesKO (*C*) mice at P30–P35 (*: p<0.05, **: p<0.01 by t-test). (*D*) TUNEL positive cells were detected in striatum and cerebellum of HTRA2 KO and NesKO brains but not in WT or NesWT littermates or the entorhinal cortex of either genotype. (*E–F*) Semi-quantitative analysis of P25–28 brains revealed a significant increase in TUNEL-positive cells in the striatum and cerebellum of HTRA2 KO (*E*) and NesKO (*F*) mice, but not in the entorhinal cortex (**: p<0.01, ***: p<0.001, by t-test). KO denotes HTRA2 KO, WT denotes HTRA2 WT, nKO denotes NesKO and nWT denotes NesWT. Scale bars; A: 500 µm, D: 100 µm.

We next examined cell death in the brain using TUNEL staining. Dying cells were detected in three discrete regions of the brain: the striatum, the cerebellum and the entorhinal cortex (Fig. 3A). Analysis revealed significant increases in the prevalence of TUNEL-positive cells in all three brain regions at P30 in both HTRA2 KO (Fig. 3B) and NesKO (Fig. 3C) animals compared to WT littermates. Cell death in the striatum and cerebellum was detectable by P25, and progressively worsens over time (Fig. 3D). Semi-quantitative analysis of P25 brains revealed significant increases in TUNEL-positive cells in the striatum and cerebellum of HTRA2 KO (Fig. 3E) and NesKO (Fig. 3F) animals compared to WT littermates, although to a lesser extent than observed at later ages. Dying cells in the entorhinal cortex were evident at P30 but not at P25, where analysis detected no significant increases in TUNEL-positive cells (Fig. 3E–F), suggesting cell death in this region may be a secondary effect of problems elsewhere in the brain. Cell death appeared to be widespread in the striatum as previously reported for mice lacking systemic HTRA2 [16]–[18]. In contrast, cerebellar cell death, which was not previously reported, was localized in the granule cell layer with a striking amount of TUNEL positive cells.

**Figure 4:**
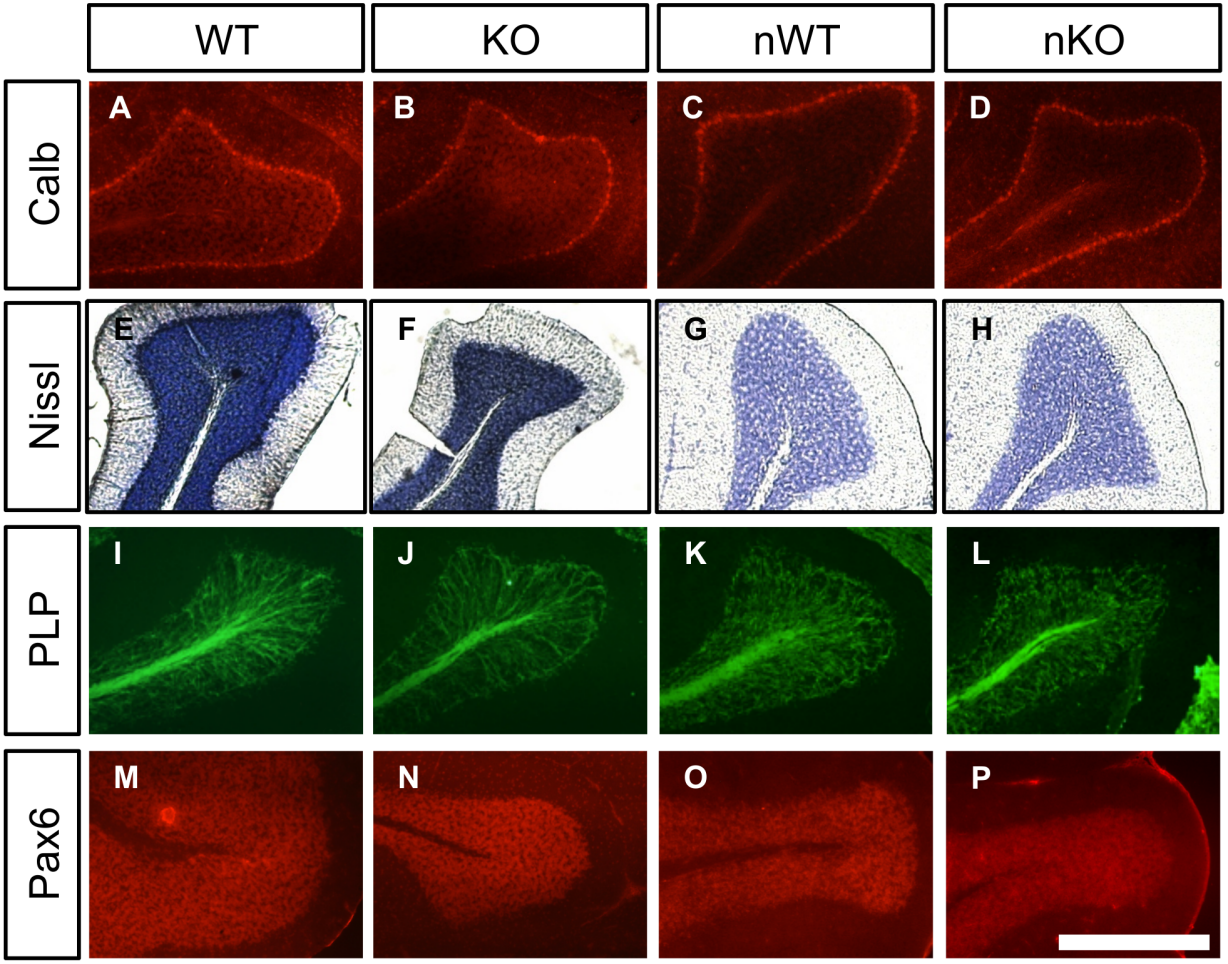
The cerebella of HTRA2 KO and NesKO mice appeared grossly normal. (*A–D*) Calbindin D-28K staining of HTRA2 WT (*A*), KO (*B*), NesWT (*C*) and NesKO (*D*) cerebellum did not reveal any differences in Purkinje cell organization in P35 cerebellum (*n* = 3). (*E–H*) Cerebellar neuronal populations did not appear altered in HTRA2 KO or NesKO mice compared to WT controls by Nissl staining at P30 (*n* = 3). (*I–L*) PLP staining for myelinated axons appeared comparable between HTRA2 KO and WT controls, and NesKO and NesWT controls at P30 (*n* = 3). (*M–P*) The cerebellar granule cell layer of P30 WT and HTRA2 KO, and NesKO and NesWT did not appear different upon staining for Pax6 (*n* = 3). KO denotes HTRA2 KO, WT denotes HTRA2 WT, nKO denotes NesKO and nWT denotes NesWT. Scale bars: A–P: 500 µm.

While TUNEL positive cells were detected in the cerebellum of every mutant mouse examined, no gross cerebellar abnormality was detected. Immunostaining for Calbindin D-28K showed a normal pattern of Purkinje cells in the HTRA2 KO and NesKO cerebella (Fig. 4A–D). Nissl staining did not reveal any differences between HTRA2 KO and WT mice, or between NesWT and NesKO mice (Fig. 4E–H). No evidence of demyelination was detected with antibody against proteolipid protein in cerebellum (Fig. 4I–L) and examining expression of Pax6, a granule layer marker, did not reveal any gross changes in the granule cell layer (Fig. 4M–P).

### Mitochondrial defects precede cell death in the cerebellum

**Figure 5:**
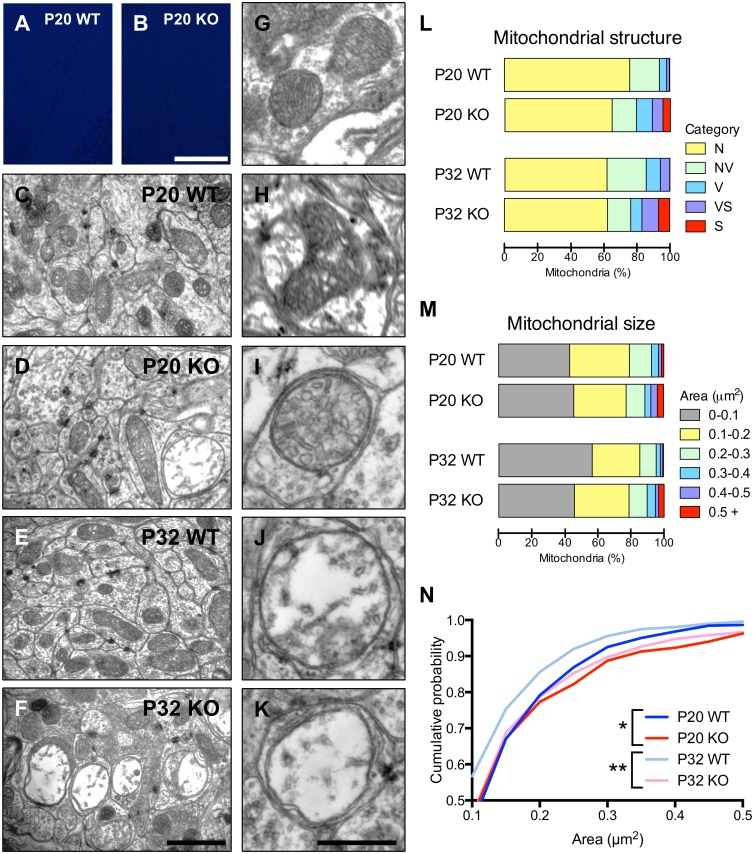
Loss of HTRA2 resulted in accumulation of large, structurally abnormal mitochondria in cerebellar granule cells. (*A–B*) TUNEL staining did not reveal any increase in cell death in the cerebellum of HTRA2 KO mice at P20. (*C–F*) Increased numbers of swollen mitochondria were detected in HTRA2 KO animals compared to WT littermates at both P20 (*C–D*) and P32 (*E–F*). (*G–K*) Five structural classes of mitochondria were detected in transmission electron micrographs of the cerebellar granule layer: N, normal (*G*), NV, normal-vesicular (*H*), V, vesicular (*I*), VS, vesicular-swollen (*J*) and S, swollen (*K*). (*L*) Mitochondria were assigned to one of the five structural classes and quantitation revealed an increase in swollen mitochondria at both P20 and P32. (*M*) The sizes of the mitochondria were measured at both P20 and P32 for both WT and HTRA2 KO animals, revealing increased proportions of large mitochondria in HTRA2 KO animals. (*N*) Calculating the cumulative probability of mitochondrial size revealed that the distributions of size were significantly altered at both P20 and P32 (*:p<0.05, **: p<0.001 by the Kolmogorov-Smirnov comparison, *n* = 1166 (P20 WT), 939 (P20 KO), 674 (P32 WT) and 1121 (P32 KO)). Scale bars: A–B: 100 µm, C–F: 1 µm, G–K: 500 nm.

Having identified cell death but no gross anomalies in the cerebellar granule layer, we examined this region more closely. Transmission electron microscopy was performed on the cerebellar granule layer prior (P20) and subsequent (P32) to the onset of cerebellar cell death (Fig. 5A–B, Fig. 3E–H). Changes in the structure of the mitochondria were detected at both age points (Fig. 5C–F), but no other striking morphological differences were observed between WT and HTRA2 KO. Quantitative analyses were performed to investigate these differences, assigning mitochondria to five structural categories (Fig. 5G–K) based on Sun et al. [37]: normal, normal-vesicular, vesicular, vesicular-swollen and swollen mitochondria. Normal mitochondria have lamellar cristae. Normal-vesicular have lamellar and vesicular structure. Vesicular mitochondria have expansions of the intracristae space. Vesicular-swollen mitochondria have vesicular morphology and regions of expanded matrix and swollen mitochondria have fewer cristae and expanded matrix space.

Compared to WT littermates, P20 HTRA2 KO mice displayed increased incidence of abnormal mitochondria with a corresponding drop in normal (0.86 times the proportion observed in WT) and normal-vesicular (0.82 times the proportion observed in WT) mitochondrial structures (Fig. 5L). There was an increase in the proportion of vesicular mitochondria evident at P20 (2.2 times the proportion observed in WT) and the amount of vesicular-swollen mitochondria was also increased (3.9 times the proportion observed in WT). The greatest increase was apparent in the proportion of swollen mitochondria observed in HTRA2 KO compared to WT controls (17.2 times the proportion observed in WT).

P32 HTRA2 KO mice displayed a larger increase in the amount of swollen mitochondria (45.3 times the proportion observed in WT) but a smaller change in the amount of vesicular-swollen mitochondria than observed at P20 (1.7 times the proportion observed in WT). While the proportion of normal mitochondria remained approximately the same as for WT at this age, the amounts of normal-vesicular and vesicular mitochondria were reduced (0.59 and 0.79 times the proportion observed in WT, respectively).

Just as with the overall phenotype of the mice, the mitochondrial phenotype was more extreme at later ages with increased incidence of swollen mitochondria. Consistent with an increased proportion of swollen mitochondria, more exceptionally large mitochondria were observed at both P20 and P32 when compared to WT littermates (Fig. 5M). The size distribution of mitochondria was significantly altered in HTRA2 KO mice at both P20 and P32 (Fig. 5N) reflecting the increased number of swollen mitochondria.

The presence of abnormally structured mitochondria at P20, while not as extreme as at P32, precedes the detectable increase in TUNEL positive cells in the cerebellar granule layer. We therefore conclude that apoptosis may be caused by impaired mitochondrial function as opposed to mitochondrial defects being a consequence of apoptosis.

### Brain-specific changes in the processing of mitochondrial fusion protein OPA1 were detected by P9

**Figure 6:**
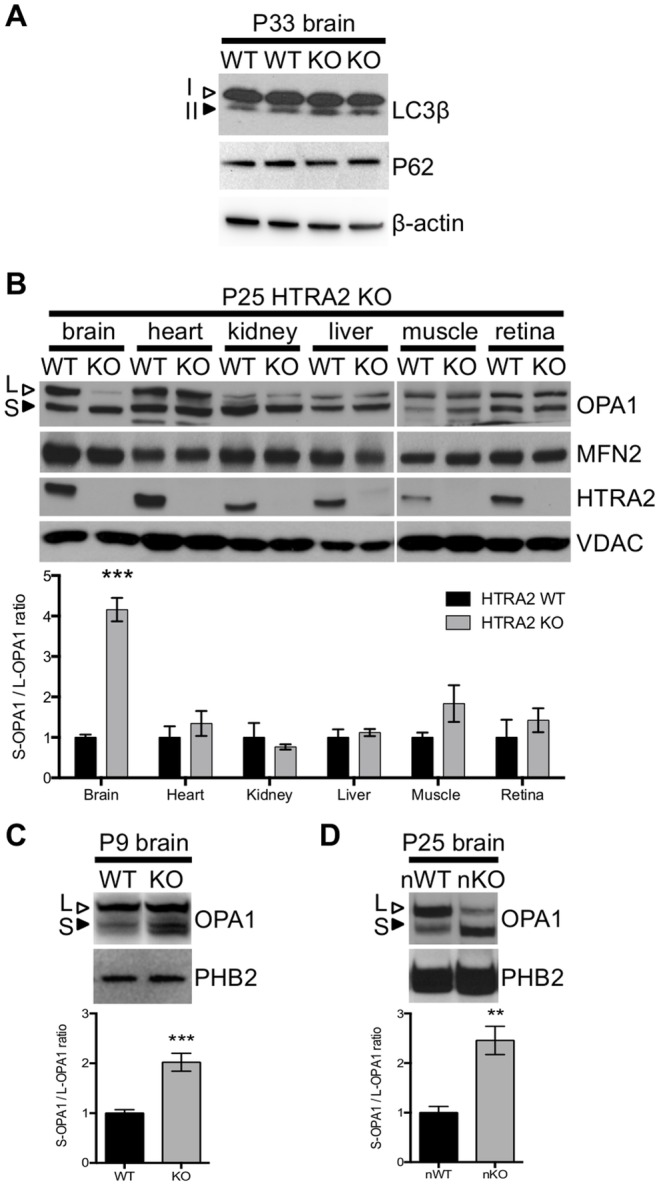
Brain-specific disruption of OPA1 processing. (*A*) Levels of autophagy markers are unchanged in P33 HTRA2 KO brain. The LC3β-II to LC3β-I ratio is unchanged in P33 brain and there is no accumulation of P62 (n = 3). Actin is included as a loading control. (*B*) Processing of OPA1 was altered in HTRA2 KO brain at P25 but not in other tissues, with increased abundance of S-OPA1 (filled arrowhead) and decreased L-OPA1 (open arrowhead). MFN2 was not altered when compared to WT. VDAC was used as a loading control. Calculating the ratio of S-OPA1 to L-OPA1 for tissues from P25–P30 HTRA2 KO mice confirmed changes in OPA1 processing were present in the brain but not in other tissues when compared to WT (***: p<0.0001 by t-test, n≥3 per genotype). (*C*) Altered processing could be detected in HTRA2 KO brain from P9 onwards. PHB2 was used as a loading control. Calculating the ratio of S-OPA1 to L-OPA1 for P9–P10 brains confirmed the change in processing (***: p<0.0001 by t-test, n≥6 per genotype). (*D*) Processing changes were also detected in NesKO brain at P25. PHB2 was used as a loading control. Calculating the ratio of S-OPA1 to L-OPA1 for brain from NesKO and NesWT mice at P24–P26 confirmed OPA1 processing was significantly altered (**: p<0.01 by t-test, n≥3 per genotype). KO denotes HTRA2 KO, WT denotes HTRA2 WT, nKO denotes NesKO and nWT denotes NesWT.

To tackle the biological processes responsible for the increased numbers of structurally compromised mitochondria in the HtrA2 KO brain, we first examined mitophagy, as inactivation of autophagy has been reported to contribute to neurodegenerative diseases including Parkinson’s disease [42]–[44]. HTRA2 has been suggested to regulate autophagy [45], we used the same marker, the mobility shift of microtubule-associated protein 1 light-chain (LC3β). In addition, we used an established method on the degradation of P62 (Sequestome-1) [46]–[52] to examine the effects of HTRA2 deficiency on this pathway. No difference was detected between the P33 HTRA2 KO and the control brains in either the relative amount of the two LC3β isoforms, LC3β-II and LC3β-I, or the abundance of P62 to indicate autophagy was involved (Fig. 6A).

We next looked into a pathway involved in mitochondrial dynamics. Since HTRA2 is located in the mitochondrial intermembrane space, we examined two membrane associated proteins: Optic Atrophy 1 (OPA1, dynamin-like 120 kDa protein) is responsible for the formation and maintenance of inner membrane cristae, and Mitofusin-2 (MFN2) is involved in regulating mitochondrial fusion in the outer membrane [22], [23], [26], [38]–[40].

Western blotting demonstrated that OPA1 processing was abnormal in the brains of HTRA2 KO mice at P25 (Fig. 6B) when compared to WT controls. We observed increased abundance of a short, processed form of OPA1 (S-OPA1) with a concomitant decrease in the long, unprocessed form (L-OPA1), and measuring the ratio of short form to long form revealed a significant increase in processing in KO brain (Fig. 6B). Interestingly, processing defects appeared to be tissue specific and were restricted to the brains of P25 HTRA2 KO mice despite the loss of HTRA2 in all tissues: while other tissues displayed different ratios of short to long forms when compared to the brain as previously described [41], no statistically significant change in processing was observed between HTRA2 WT and KO samples except for in brain (Fig. 6B). This change in OPA1 processing could be detected as early as P9 (Fig. 6C). OPA1 processing was also found to be abnormal in the brain of P25 NesKO animals (Fig. 6D).

In contrast to the observed changes in OPA1 processing, no change in the level of MFN2 was detected between mutant and control in any tissue at P25 (Fig. 6B). These data are consistent with the electron microscopy results that revealed abnormalities in inner membrane architecture but not outer membrane organization (Fig. 5D, F).

Since alteration of the ratio of L-OPA1 to S-OPA1 is sufficient to cause disorganization of the cristae in mitochondria [41], our observations of the tissue specificity and temporal prevalence suggest that the presence of HTRA2 maintains proper OPA1 processing, whether directly or indirectly through preventing mitochondrial stress, regulating mitochondrial structures in the brain.

### Electron transport chain enzymes are compromised in HTRA2 KO brains

Considering the inner mitochondrial membrane is the site of oxidative phosphorylation in mitochondria, and this is the site of morphological disruption in the brains of HTRA2-deficient mice, we next investigated respiratory function within the HTRA2 KO brain. Electrons can enter the oxidative phosphorylation chain via either Complex I (NADH dehydrogenase) using NADH as an electron donor, or via Complex II (succinate dehydrogenase) using FADH as a donor. Electrons are then passed along the electron transport chain, generating a proton gradient required to drive ATP synthesis.

**Figure 7:**
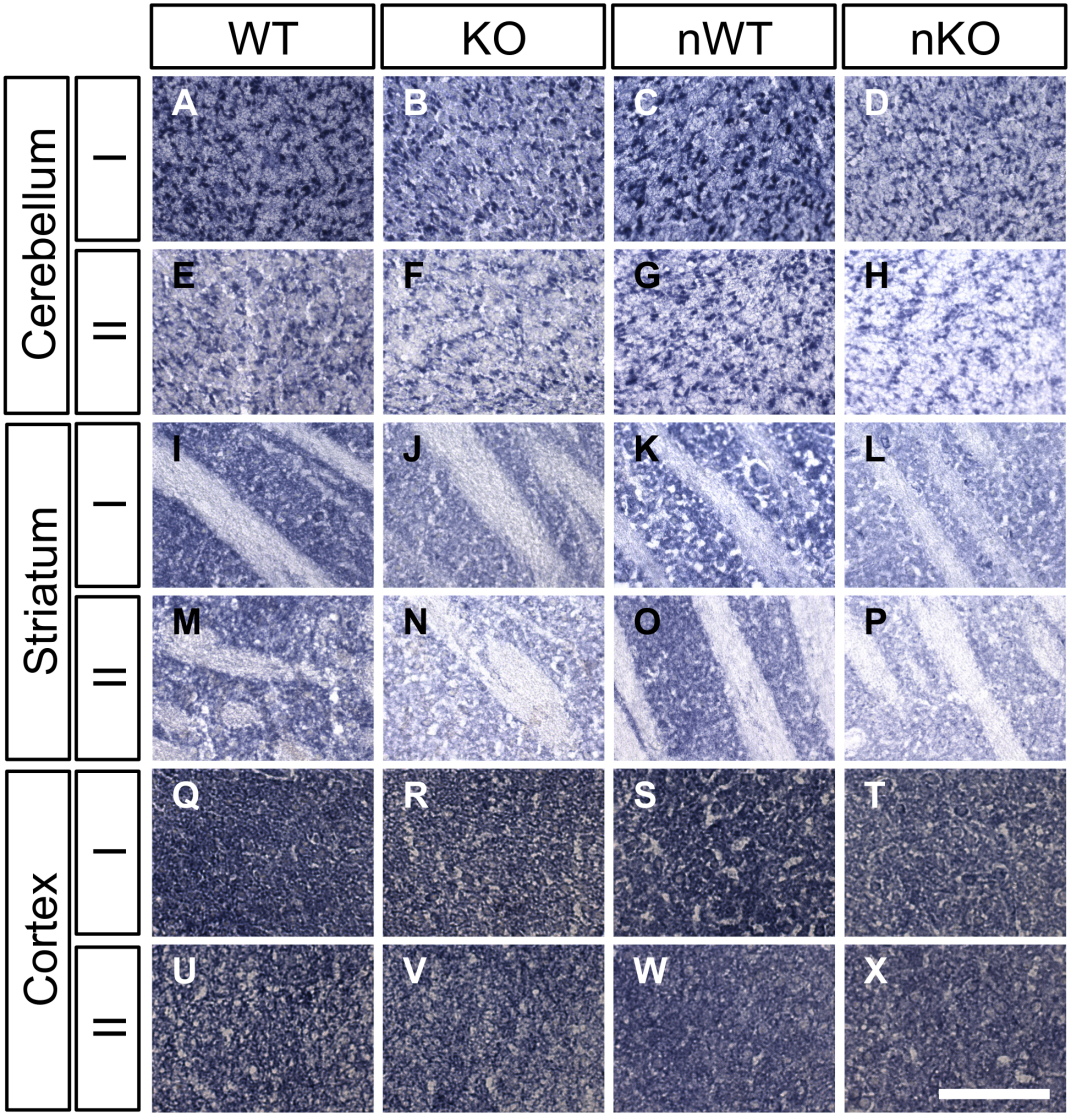
Respiratory complex levels are reduced in HTRA2-deficient brain in a region-specific manner. (*A–X*) Histochemical staining for Complex I (*A–D, I–L, Q–T*) and Complex II (*E–H, M–P, U–X*) revealed decreased enzyme levels in the cerebellum (*A–H*) and striatum (*I–P*), but not in the cerebral cortex (*Q–X*), of P25 HTRA2 KO and NesKO animals when compared to WT and NesWT controls (n≥3 per genotype). KO denotes HTRA2 KO, WT denotes HTRA2 WT, nKO denotes NesKO and nWT denotes NesWT. Scale bar: A–X: 100 µm.

We used a previously described *in* *situ* histochemical method [32] to assay Complex I and Complex II in P25 brains ofHTRA2-deficient mice. The levels of Complex I and Complex II were reduced in the cerebellar granule cell layer of HTRA2 KO and NesKO individuals compared to WT littermates (Fig. 7A–H). A similar reduction was observed in the striatum (Fig. 7I–P) although levels elsewhere in the brain, for example in the cerebral cortex, did not appear to be affected (Fig. 7Q–X). Interestingly, the affected areas correlate with the observed regions of cell death in HTRA2-deficient brain. These data suggest that electron transport chain function is compromised in HTRA2 KO and NesKO brain, likely as a result of the morphological abnormalities, and seemingly in a region-specific manner.

## Discussion

### Neural deletion of *Htra2* is sufficient to cause all phenotypes observed upon systemic knockout

Systemic deletion or mutation of HTRA2 in mice causes neurological phenotypes, lymphopenia and a failure to thrive [16]–[18]. Neural expression of *Htra2* in a HTRA2-deficient background prevents the development of these phenotypes but reveals accelerated aging in peripheral tissues [19], suggesting that these tissues might contribute to phenotypes in systemic knockouts. Here we demonstrate that Cre-mediated deletion of *Htra2* from the nervous system is sufficient to induce the phenotypes of a complete knockout of the gene, including failure to gain weight and atrophy of the thymus and spleen, despite normal expression of HTRA2 in these immune organs. These results suggest that the observed effect is not cell intrinsic but instead relies on input from the nervous system.

We postulate that the importance of HTRA2 in neuronal cells is related to the brain-specific change in OPA1 cleavage. Several isoforms of OPA1 exist, and these can be further processed to generate 6 distinct forms of the protein [26]–[28], [38], [41]. It is not yet clear why changes in OPA1 processing are restricted to the brain. The fact that the retina is comprised of neurons and yet aberrant processing is not detected could be due to a difference in relative importance of isoforms between retina and brain, although isoform 1 was reported to be the primary, most abundant form in both tissues [41]. Alternatively, the lack of changes in processing in retina may suggest the requirement for an additional brain-specific factor, a specific neuronal cell type or connection, or a cell environment not found in the retina.

It is likely that there are further brain-specific changes involved in the development of phenotypes in mice lacking HTRA2 in neuronal populations – for example, the transcription factor CHOP is known to be upregulated as part of a brain-specific integrated stress response [53]. Identification of brain-specific changes will help explain the importance of HTRA2 in neural tissues and may eventually point to potential sites of intervention for therapeutic purposes.

### The cerebellum is a novel site of interest in *Htra2*-deficient mice

Previous examination of both *Htra2^mnd2^* and *Htra2^tm1Jdo^* mice have reported parkinsonian symptoms and increased death in the striatum [16], [18], but whether there exists cerebellar pathology was not mentioned. Here we identify two additional regions of cell death identified by increased TUNEL staining: the granule layer of the cerebellum and the entorhinal cortex. Further, the onset of cell death in striatum and cerebellum occur earlier, while cell loss in the entorhinal cortex can only be detected at later ages, suggesting the latter may be a consequence of insults to other brain regions. In contrast, like previous reports [8], [9], we did not detect neuronal cell loss in the Substantia nigra.

Interestingly, while defects in the striatum have been well documented in PD structural changes in the cerebellum have also recently been implicated in PD patients [54]–[58]. In particular, cerebellar pathology appears to be more prominent in tremor-dominant PD patients. The cerebellar structure and their reciprocal connections to the basal ganglia also may contribute significantly to the signs and symptoms of PD. While the cerebellum related pathology is difficult to recognize in PD patients, both mice and monkeys treated with MPTP showed signs of cell degeneration in the cerebella [59]–[66]. The cell death detected in the cerebellum of HTRA2 KO and NesKO mice may be instrumental in the development of those phenotypes that resemble symptoms of PD.

Prior to cell death in the granule cell layer of the cerebellum, mitochondrial morphology is compromised. Previous studies on HTRA2-deficient MEF cells were conflicting in their accounts of the structural integrity of mitochondria [29], [67]. Here we provide *in* *vivo* evidence that the absence of HTRA2 leads to accumulation of swollen mitochondria with loss of inner membrane architecture. This increase in abnormal mitochondria is associated with increased processing of OPA1, where increased levels of S-OPA1 are detected alongside a decrease in the abundance of L-OPA1.

Disrupted OPA1 processing causes fragmentation of the mitochondrial network and disorganization of cristae structure [22], [24], [68]. However, dysfunction of mitochondria accompanied by decreased mitochondrial ATP, changes in mitochondrial membrane potential or loss of mitochondrial DNA could also instigate changes in OPA1 processing. As HTRA2 deficiency has previously been reported to cause reduction in mitochondrial membrane potential, reductions in mitochondrial ATP concentrations and mitochondrial DNA deletions [17], [19], [67], [69]–[71] it is not yet clear whether the loss of HTRA2 protein is a direct cause of aberrant OPA1 processing or whether the effect is an indirect consequence of mitochondrial stress.

The disruption of the mitochondrial inner membrane is associated with compromised function in the striatum and cerebellum of HTRA2-deficient mice, as assayed by histochemical staining for Complex I and Complex II. It is especially intriguing that this reduction in the levels of respiratory enzymes occurs in a region-specific manner and is confined to the regions that undergo cell death. Considering this reduction is relatively extensive compared to the modest number of dying cells in these regions, it may be that impaired mitochondrial function contributes to cell death.

These data suggest that damaged mitochondria trigger cell death rather than being degraded by the autophagy system. Both HTRA2 and OPA1 have been implicated in the control of mitochondrial functions and autophagy [19], [45], [72], [73]; the loss of HTRA2 may cause a decrease in mitophagy and reduce the clearance of compromised mitochondria. However, preliminary studies on HTRA2 KO brain did not demonstrate any changes in the LC3β-II/LC3β-I ratio or reveal accumulation of P62. A detailed, dynamic analysis of autophagy in cells lacking HTRA2 would be beneficial in fully answering this question, and further studies are warranted to examine how the cell deals with compromised mitochondria.

### HTRA2-deficient mice are compromised as early as P7

Changes in OPA1 processing within the brain can be detected as early as P9. Even earlier than this change, we could detect phenotypes in mice lacking HTRA2. Neonatal behavioral testing of both HTRA2 KO and NesKO mice provided evidence supporting our hypothesis that a subtle phenotype is present in HTRA2 deficient mice beginning early in development. Specifically, data from the hind-limb suspension test showed that, beginning at approximately P9, HTRA2 deficient pups exhibit a consistent deficit in hang time and in the number of hind limb pull attempts when compared to WT pups. Additionally by P19, close to the time when the failure to thrive begins, HTRA2-deficient pups display markedly reduced motor activity when compared to WT pups demonstrating that an observable motor phenotype is present before the development of an ataxic gait.

Using this new floxed allele to control the timing of deletion may reveal new insights into the mechanism and kinetics of disease, and the relative importance of HTRA2 at different developmental time points. It will allow the fine query of the role of HTRA2 in individual cell types and how these cells interact with a normal environment. The allele will enable increasingly directed deletion of *Htra2*, for example specifically deleting in the cerebellum, allowing systematic examination of the effect of specific loss of HTRA2 on phenotype development. Analysis of such deletions will provide further insight into the etiology of disease in these models, with the ultimate goal of developing targeted interventions and treatment regimens.

### Conclusion

The absence of the mitochondrial protein HTRA2 in the nervous system is sufficient to generate the neurological and immunological defects observed in systemically deleted mice. Further, our results demonstrate for the first time that mitochondrial anomalies in the cerebellum are an early phenotype of *Htra2* mutant mice that precede the onset of parkinsonian symptoms. Future studies are needed to determine whether *HTRA2* mutations lead to the cerebellar pathology in Parkinson’s disease patients. If confirmed, these findings may lead to earlier diagnosis and better treatment of this disorder.
